# Emotional shaping of spatial encoding along the dorsal ventral axis of the hippocampus

**DOI:** 10.3389/fnsys.2026.1888312

**Published:** 2026-07-03

**Authors:** Azul Silva, Gabrielle Girardeau

**Affiliations:** 1 Center of Neuroscience Neuro-SU, CNRS, Inserm, Sorbonne Université, Paris, France; 2 CNRS, Inserm, Institut de Biologie Paris-Seine, IBPS, Sorbonne Université, Paris, France

**Keywords:** dorsal hippocampus, ventral hippocampus, emotional coding, spatial coding, valence

## Abstract

Emotional memories are essential for survival, enabling individuals to avoid future threats or seek safety or food. How does the brain link a specific spatiotemporal context with the associated emotional experience? The hippocampus (HPC) encodes spatial information through place cells, which collectively form a cognitive map that adapts to contextual changes. Along its longitudinal axis, the HPC shows marked functional divergence: the dorsal HPC supports fine-tuned spatial representations, whereas the ventral HPC exhibits broader connectivity with limbic circuits and plays a key role in encoding emotional valence, salience, anxiety, and motivational state. Here, we synthesize current evidence comparing spatial and emotional coding across these regions, emphasizing differences in spatial properties and circuit interactions. Finally, we propose a framework describing how dorsal and ventral HPC integrate spatial and emotional information to guide adaptive behavior.

## Introduction

Emotional memories are essential for survival, enabling individuals to avoid future threats and potentially life-threatening situations, as well as to return to locations where food or shelter were previously found. Memory-guided behavior relies on internal representations of both salient emotional experiences and the spatial contexts in which they occur. A central question, therefore, is how the brain links the spatiotemporal context and the emotion experienced within it. The hippocampus constitutes a key component of the neural network supporting this integrative process.

In rodents, hippocampal research has mainly focused on the dorsal region (dHPC) since the discovery of place cells, pyramidal neurons that exhibit location-specific firing, defined as their place field (O’Keefe, 1990; O’Keefe and Dostrovsky, 1971). As a population, place cells form cognitive maps (Tolman, 1948) that support spatial learning. In response to contextual changes, place cells reorganize their firing properties, a process known as remapping (Bostock et al., 1991; Muller et al., 1991). Exposure to novel environments induces a drastic reorganization of the hippocampal cognitive map (global remapping) (Muller and Kubie, 1987), whereas slighter changes produce more subtle effects: firing rate changes (rate remapping), and/or shifts in place fields location (partial remapping; Kubie et al., 2020).

During the exploration of an environment, the hippocampal local field potential (LFP) is dominated by 6–12 Hz theta oscillations (Buzsáki, 2002). As an animal crosses a place field, the corresponding place cell fires at progressively earlier phases of the theta cycle, reaching the trough when the animal is at the center of the field. At the population level, this temporal shift implies that a single theta cycle contains sequential information reflecting the animal’s past, present, and future positions. This phenomenon, known as phase precession (O’Keefe and Recce, 1993), organizes place cell activity into theta sequences that operate on a timescale well-suited for synaptic plasticity and neuronal ensembles formation (Drieu and Zugaro, 2019; Foster and Wilson, 2007).

The hippocampus exhibits pronounced heterogeneity along its dorsoventral axis, reflected in both its anatomical connectivity and functional specialization (Bannerman et al., 2004; Fanselow and Dong, 2010; Kheirbek et al., 2013; Strange et al., 2014). The dHPC is primarily linked to spatial processing, whereas the ventral hippocampus (vHPC) also encodes emotion-related information. Unlike the dHPC, the vHPC has reciprocal anatomical connections with the brain’s emotion-processing network. Given its distinct connectivity and limited spatial coding, the vHPC may be suited to encode the emotional context of experiences.

The aim of this mini-review is to provide a detailed characterization of the emotional and spatial coding in the vHPC, highlighting both its differences and points of convergence with the dHPC. In doing so, we outline a conceptual framework for how these two regions may interact during emotional processing.

## Coarser vs precise spatial coding

**FIGURE 1:**
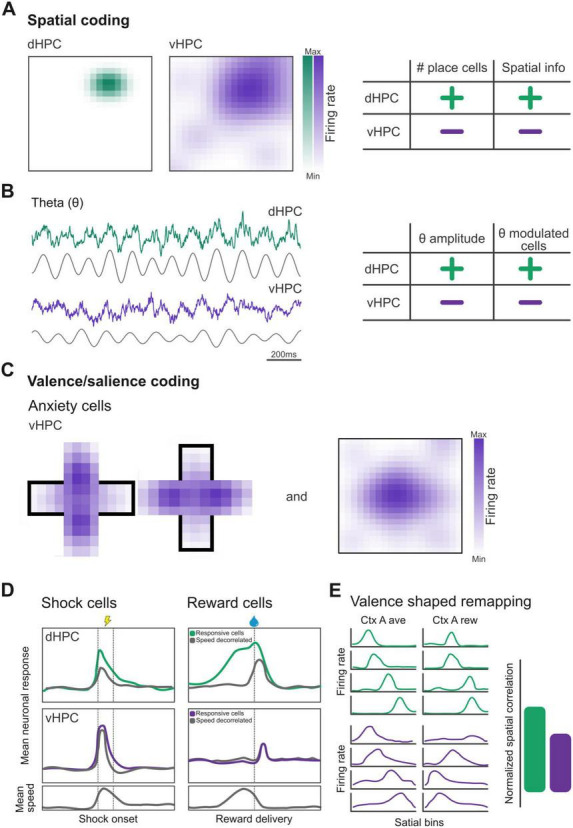
Spatial and valence/salience coding along the dorsal-ventral hippocampal axis. **(A)** Schematic comparison of spatial coding properties. dHPC place cells (green) exhibit sharp, well-defined place fields, whereas the vHPC (purple) contains fewer place cells with larger and less precise fields. **(B)** Example local field potential **(LFP)** theta oscillations recorded from the dHPC (green) and vHPC (purple) during movement. The grey trace represents the LFP filtered in the theta frequency band (5–10 Hz). Theta amplitude and the proportion of theta-modulated cells are lower in the vHPC than the dHPC. **(C)** Schematic representation of vHPC anxiety-cells. These neurons fire consistently in anxiogenic locations, such as the open arms of an elevated plus maze, even when the maze is rotated, and also in the center of an open field. **(D)** Schematic of shock- and reward-responsive cells. Note that dHPC activity is strongly modulated by locomotor variables such as speed, whereas vHPC activity shows weaker modulation. **(E)** Schematic comparison of dorsal (upper panel) and ventral (lower panel) place cells in context A under aversive (shock-associated) and rewarding conditions. vHPC place cells exhibit lower spatial correlation across contexts, reflecting greater remapping. Adapted from Morici et al. (2026).

The vHPC, like the dHPC, contains place cells, but in smaller proportions and with larger, more overlapping place fields (Jung et al., 1994; Kjelstrup et al., 2008). vHPC place cells also remap in response to changes in the physical environment (Keinath et al., 2014). Overall, spatial representations in the vHPC are coarser, less spatially precise, and of reduced dimensionality (Chockanathan and Padmanabhan, 2021), whereas the dHPC maintains a high-resolution map of the environment characterized by sharp, non-overlapping place fields (O’Keefe, 1990, 1976; Figure 1A).

The vHPC shows theta oscillations during movement and REM sleep, but they are less regular and have substantially lower power, amplitude, and proportion of theta-locked neurons than in the dHPC (Patel et al., 2012; Royer et al., 2010; Figure 1B). In the dHPC, theta strongly correlates with locomotion variables, such as running speed or acceleration, whereas this correlation is weaker in the vHPC (Kropff et al., 2021; Patel et al., 2012). Phase precession is also present in the vHPC, but slower (Kjelstrup et al., 2008). As a result, it maintains a temporally compressed representation of trajectories, but over a considerably larger spatial scale. Theta oscillations travel along the dorso-ventral axis like a wave, maintaining coherence with a gradually increasing phase shift from the dorsal to the ventral pole (Lubenov and Siapas, 2009; Patel et al., 2012).

Collectively, the coding properties of the vHPC suggest that, while it maintains a framework analogous to the dHPC, the vHPC is better suited to integrating coarser contextual information than precise spatial coding. This broader representational structure may facilitate the integration of emotionally salient information.

## Broad emotional coding vs emotionally modulated spatial maps

Extensive evidence demonstrates that spatial representations in the dHPC can be modulated by the salience or emotional valence of an experience. Following exposure to an aversive event, such as a footshock or predator odor, place cells undergo remapping even in the absence of any physical changes to the environment (Moita et al., 2004; Okada et al., 2017; Schuette et al., 2020; Wang et al., 2012). In addition, place fields often shift toward locations associated with positive outcomes (goal-directed remapping) or toward sites linked to negative stimuli, resulting in a higher concentration of place fields around emotionally salient regions (Dupret et al., 2010; Hollup et al., 2001). Moreover, a subset of dHPC neurons is selectively activated at reward sites regardless of their spatial position, thereby encoding positive valence independently of spatial context (“reward cells”) (Gauthier and Tank, 2018). Additional populations of neurons are specifically active during freezing (Schuette et al., 2020). Together, these results show that dHPC can incorporate valence-related information into its spatial map, marking the locations of both rewards and threats while retaining spatial precision.

A growing body of evidence indicates that emotional modulation is even more pronounced in the vHPC, likely arising from its bidirectional connectivity with multiple cortical and subcortical regions involved in emotional processing, including anxiety-like behavior. Subsets of vHPC neurons exhibit a similar activity increase in different anxiogenic locations, such as the open arms of an elevated plus maze or the center of an open field (Ciocchi et al., 2015; Forro et al., 2022; Jimenez et al., 2018; Figure 1C). These anxiety-related neurons show minimal overlap with classical place cells (Ciocchi et al., 2015), suggesting that the vHPC generalizes across anxiogenic situations independently of their specific locations. In addition, modulating vHPC theta power modifies the amount of exploration of anxiogenic zones associated with predator odor (Mikulovic et al., 2018). Population activity within the ensemble of anxiogenic cells scales monotonically with the level of environmental anxiety (Cerquetella et al., 2025), and vHPC activity immediately before entering an anxiogenic zone predicts subsequent exploration (Malagon-Vina et al., 2023). This activity is regulated by an inhibitory microcircuit involving parvalbumin interneurons (Forro et al., 2022; Volitaki et al., 2024). Anxiety-related neurons preferentially project to the lateral hypothalamus (LH) and medial prefrontal cortex (mPFC), but not the baso-lateral amygdala (BLA; Ciocchi et al., 2015; Padilla-Coreano et al., 2016). Optogenetic inhibition of the vHPC or its projections reduces anxiety-related behavior, whereas optogenetic activation enhances it (Cerquetella et al., 2025; Jimenez et al., 2018; Padilla-Coreano et al., 2016). Thus, the vHPC supports anxiety-related behaviors via interneuron-mediated local processing and projections to the LH and mPFC.

The vHPC is also critically involved in fear processing. Temporary inactivation or lesions disrupts both cued and contextual fear learning (Bast et al., 2001; Maren and Holt, 2004). A subset of vHPC neurons responds robustly to aversive stimuli, such as footshocks or eyelid shocks (Figure 1D; Herbst et al., 2022; Jimenez et al., 2020; Morici et al., 2026), and these shock-responsive neurons preferentially project to the BLA (Jimenez et al., 2020). Notably, fear-related behaviors are regulated by a distinct inhibitory microcircuit, involving mainly vasoactive intestinal peptide and somatostatin interneurons, which differs from the circuit implicated in anxiety (Li et al., 2024). The dHPC and vHPC contribute differently to fear learning: during cued fear conditioning, vHPC activity is more closely related to the acquisition of the conditioned fear (CS-US association), while dHPC neural responses are more prominent during freezing (Pompili et al., 2026), suggesting that vHPC is implicated in the representation of external cue-outcome association. In contrast, during both aversive and appetitive conditioning, similar activity patterns have been reported in the dHPC and vHPC during the interval between CS and US presentation, indicating a shared anticipatory coding across the dorsoventral axis. Notably, this anticipatory response is weak when the aversive stimulus is inescapable, but is strongly expressed when animals can avoid it through an instrumental action, highlighting the role of behavioral relevance in shaping this coding (Biane et al., 2023). Beyond fear conditioning, vHPC also participates in pain processing (Ma et al., 2019; Shao et al., 2023). Exposure to noxious stimuli suppresses the activity of a specific subpopulation of vHPC neurons, and optogenetic manipulation of this ensemble bidirectionally modulates pain-related behaviors. Its inhibition exacerbates nociceptive responses, whereas its activation produces analgesic effects (Shao et al., 2023). Both vHPC-BLA and vHPC-infralimbic cortex are involved in chronic inflammatory pain, but at different time stages. Together, these results suggest that the vHPC supports a flexible, context-dependent representation of aversive experiences, coding aversive stimulus, cue–outcome associations, anticipatory signals, and pain processing.

Reward also shapes stimulus representations in the vHPC. The overrepresentation of high-value reward locations increases from dorsal to intermediate hippocampus (Jin and Lee, 2021). While the dHPC tends to recruit distinct neuronal populations for different reward locations that remap with changes in learned reward locations, neurons in the intermediate hippocampus often respond to multiple reward sites (Jarzebowski et al., 2022). This indicates a shift along the dorsoventral axis toward reward representations that are less tightly anchored to specific spatial locations and more generalizable across rewards. In this line, vHPC place cells present higher remapping than dHPC ones in response to changes in the contextual emotional valence, for example, when a previously rewarded environment becomes aversive (Figure 1E; Morici et al., 2026), suggesting that vHPC is more sensitive than dHPC to contextual valence. Consistent with this view, over the course of learning, vHPC neurons show larger changes in their responses to neutral stimuli that predict rewards than neurons in the dHPC (Biane et al., 2023). The vHPC, as the dHPC, interacts with the nucleus accumbens (Nac) during goal-directed behaviors (Ciocchi et al., 2015; Ito and Lee, 2016; Patterson et al., 2025; Sosa et al., 2020; Wee et al., 2024). Notably, vHPC projections to the NAc are more prominent than those from the dHPC, which are relatively sparse (Brog et al., 1993; Li et al., 2018). Moreover, the vHPC predominantly targets the NAc shell, and this pathway has been extensively implicated in hedonic processing (Bagot et al., 2015; LeGates et al., 2018; Patterson et al., 2025; Pignatelli et al., 2021). Distinct vHPC subpopulations show reward-zone–related responses, either increasing or decreasing their firing as animals approach a reward location. Excited neurons are enriched among vHPC projections to both the PFC and the NAc, whereas inhibited neurons primarily target the NAc (Ciocchi et al., 2015). Consistent with these findings, Iyer et al. showed that vHPC glutamatergic neurons that project to the NAc encode reward-related information via suppression of their neuronal activity, particularly following unrewarded trials (Iyer et al., 2025). This pathway has also been implicated in food-related behaviors (Décarie-Spain et al., 2025; Reed et al., 2018; Wee et al., 2024). vHPC–NAc–projecting neurons are bidirectionally modulated during food investigation and consumption: they increase their firing during investigation, and they are rapidly inhibited upon the initiation of eating (Wee et al., 2024). Optogenetic inhibition of vHPC-to-NAc-projecting neurons correspondingly increases food consumption (Reed et al., 2018). This circuit also assigns an hedonic value to food: low-frequency stimulation of vHPC terminals in the NAc during consumption enhances palatability (Yang et al., 2020). Overall, the vHPC robustly encodes reward-related information, integrating contextual, predictive, and hedonic signals to guide goal-directed behavior.

Social interactions, depending on the nature of the encounter, could be a rewarding or aversive experience. The vHPC has been implicated in different aspects of social behavior, like social interaction and social memory. vHPC neurons respond to the presence of conspecifics (Rao et al., 2019), showing even stronger responses than in the dHPC (Rao et al., 2019; Wu et al., 2023). In contrast to pyramidal neurons, vHPC parvalbumin interneurons show greater activation in response to a novel conspecific than to a familiar one (Deng et al., 2019). Within the vHPC, distinct neuronal populations encode conspecific identity and social properties such as sex or strain (Watarai et al., 2025). This response is at least partially mediated by the dorsal CA2 and the medial amygdala, as their ablation disrupted the population coding of sex (Watarai et al., 2025). Dorsal CA2, which has been extensively implicated in social behaviors (Oliva, 2022), projects to the vHPC, forming an intra-hippocampal circuit necessary for social memories (Meira et al., 2018). Recently, ventral CA2 has also been linked to social behaviors such as aggression, although, unlike its dorsal counterpart, it does not appear to support social memory (Boyle et al., 2025). On the other hand, vHPC CA1 is implicated in social memory, enabling discrimination between novel and familiar mice or between neutral and social-rewarding contexts (Duarte et al., 2024; Okuyama et al., 2016). Notably, a large proportion of cells active in socially rewarding contexts also respond to other positive stimuli, such as sucrose and female conspecifics, but not to stimuli of opposite valence (Duarte et al., 2024), suggesting a certain degree of generalization across stimuli sharing the same emotional valence. The vHPC encoding of social and socio-emotional stimuli involves several extra-hippocampal pathways. Optogenetic inhibition of the vHPC projections to the NAc, but not to the BLA, reduced discrimination between familiar and novel mice (Okuyama et al., 2016). In addition, projections from the locus coeruleus to the vHPC modulate social-contextual representations (Duarte et al., 2024). The vHPC–medial prefrontal cortex pathway is also involved in social behavior. This projection is hyperactive in a mouse model of autism spectrum disorder characterized by social deficits, and prolonged excitation of this pathway in wild-type mice disrupts social memory (Phillips et al., 2019).

Through both correlational and causal approaches, these studies collectively demonstrate that the vHPC plays a central role in behaviors driven by diverse forms of emotional valence and salience. Moreover, the vHPC may implement a degree of functional segregation across its local inhibitory microcircuits and its projection pathways, with distinct circuits preferentially supporting positive or negative valence processing, although these circuits do not exhibit strictly fixed functional roles. For example, distinct reward- and aversive-related engrams have been identified within the vHPC, with aversive ensembles enriched in BLA-projecting neurons and rewarding ensembles enriched in NAc-projecting neurons (Shpokayte et al., 2022). Despite this segregation, both vHPC–BLA and vHPC–NAc pathways can switch between driving either preference or avoidance, revealing substantial plasticity in these circuits. Indeed, a more recent study showed that vHPC populations maintain stable representations of a stimulus even when its associated valence is reversed. This also challenges the valence-centric view of vHPC coding by suggesting that vHPC rather encodes stimuli according to their identity and salience (Biane et al., 2025).

Consistent with this role in salience processing, the vHPC has also been implicated in novelty detection. Exposure to a novel context increases vHPC theta power and weakens vHPC–mPFC functional connectivity, facilitating strategy switching in a subsequent alternation task. As animals habituate to the novel environment, theta power decreases and vHPC–mPFC connectivity strengthens (Park et al., 2021). Novelty processing also engages intra-hippocampal communication: ventral mossy cells transmit novelty-related signals to the dHPC, revealing a dorsoventral mechanism that gates the detection and integration of environmental novelty (Fredes et al., 2021).

## Discussion

The hippocampus is a heterogeneous structure exhibiting distinct patterns of connectivity and functional specialization along its dorsoventral axis. The dHPC maintains a highly precise spatial map that can incorporate information about the locations of both positive and negative events. Place cells in the dHPC remap in response to various aversive and rewarding stimuli, consequently embedding valence-related information within a spatially anchored framework. In contrast, vHPC exhibits a coarser spatial map characterized by larger and sparser place fields supporting a broader, context-dependent form of coding encompassing behavioral states and subjectively salient stimuli. Importantly, this coding is supported by partially distinct neural pathways within the vHPC that differentially regulate anxiety, fear, and reward-related behaviors. The coexistence of these two types of representations may allow the hippocampus to simultaneously preserve spatial accuracy and behavioral relevance, linking the location of events with their motivational and emotional significance.

Neuronal assemblies that include neurons from both dHPC and vHPC were recently identified during fear conditioning, revealing synchronization along the dorsal-ventral axis (Pompili et al., 2026). These joint assemblies recruit neurons that respond to different aspects of fear-related behavior in each region (Pompili et al., 2026). These assemblies also disambiguate an aversive experience from a rewarded one better than assemblies containing members from a single region (Morici et al., 2026). Through this coordinated activity, distributed representations across the dorsoventral axis may bind contextual, spatial, and emotional information during awake encoding. Theta oscillation remains relatively synchronous along the septotemporal axis, which may be one of the substrates for functional communication between both poles. A principal anatomical substrate for intra-hippocampal longitudinal integration lies within CA3, which exhibits extensive associative projections along the septotemporal axis (Ishizuka et al., 1990).

A remaining open question is how these distributed representations are transformed into long-term memories after the emotional experience. Hippocampal sharp-wave ripples (SWRs) and associated reactivation are thought to support memory consolidation during sleep (Buzsáki, 1998; Ego-Stengel and Wilson, 2010; Girardeau et al., 2009; Pavlides and Winson, 1989). Both dHPC and vHPC generate SWRs during non-REM sleep, and a subset of these events occurs in a coordinated manner across the two regions (Morici et al., 2026; Patel et al., 2012). Coordinated SWRs have been shown to sustain joint dorsoventral assemblies reactivations (Morici et al., 2026), providing an ideal time window for binding precise spatial and emotional offline representations. Dorsal and ventral SWRs also support the coordination of the hippocampus with other cortical and subcortical structures (Girardeau et al., 2017; Gomperts et al., 2015; Jadhav et al., 2016; Miyawaki and Mizuseki, 2022; Rothschild et al., 2017; Sosa et al., 2020). Future studies will need to establish how intra-hippocampal and inter-regional coordination during sleep supports the consolidation of long-term memories that integrate spatial and salience/emotional-related representations.
